# Light-intensity physical activity and mental ill health: a systematic review of observational studies in the general population

**DOI:** 10.1186/s12966-021-01196-7

**Published:** 2021-09-15

**Authors:** Mireia Felez-Nobrega, Judit Bort-Roig, Ruimin Ma, Eugenia Romano, Matthew Faires, Brendon Stubbs, Emmanuel Stamatakis, Beatriz Olaya, Josep Maria Haro, Lee Smith, Jae Il Shin, Min Seo Kim, Ai Koyanagi

**Affiliations:** 1 Research and Development Unit, Parc Sanitari Sant Joan de Déu, C/ Dr. Antoni Pujadas 42, 08830 Sant Boi de Llobregat, Barcelona Spain; 2 Centre for Biomedical Research on Mental Health (CIBERSAM), Madrid, Spain; 3 Sport and Physical Activity Research Group and Centre for Health and Social Care Research, Department of Physical Activity and Sports Sciences, University of Vic - Central University of Catalonia, Vic, Barcelona Spain; 4 Institute of Psychiatry, Psychology and Neuroscience, King’s College London, De Crespigny Park, London, SE5 8AF UK; 5 South London and Maudsley NHS Foundation Trust, Denmark Hill, London, SE5 8AZ UK; 6 Charles Perkins Centre, School of Health Sciences, Faculty of Medicine and Health, University of Sydney, Sydney, NSW Australia; 7 The Cambridge Centre for Sport and Exercise Sciences, Anglia Ruskin University, Cambridge, UK; 8 Department of Pediatrics, Yonsei University College of Medicine, Seoul, 03722 Republic of Korea; 9 Korea University, College of Medicine, Seoul, Republic of Korea; 10 Genomics and Digital Health, Samsung Advanced Institute for Health Sciences and Technology (SAIHST), Sungkyunkwan University, Seoul, Republic of Korea; 11 ICREA, Pg. Lluis Companys 23, Barcelona, Spain

**Keywords:** Mental, Depression, Physical activity, Prevention, Public health

## Abstract

**Background:**

Most of theevidence has focused on examining the influence of moderate-to-vigorous intensity physical activity on mental health, but he role of light intensity physical activity (LIPA) is less understood. The purpose of this systematic review was to assess the relationship between time spent in LIPA and mental ill health across the lifespan.

**Methods:**

Data were obtained from online databases (Medline, Embase, Scopus, PsychInfo and CINAHL). The search and collection of eligible studies was conducted up to May 28, 2020. Observational studies conducted in the general population and reporting on the association between LIPA (1.6–2.9 metabolic equivalents; either self-reported or device-based measured) and mental ill health were included.

**Results:**

Twenty-two studies were included in the review (16 cross-sectional and 6 longitudinal). In older adults (≥ 65 years) and adults (18–64 years), the evidence examining the relationship between LIPA and depressive symptoms is mixed. Data on anxiety, psychological distress and overall mental health are scarce, and results are inconclusive. There is no evidence suggesting favorable associations between LIPA and anxiety in college students. Finally, very limited data was found in adolescents (11–17 years) (*n* = 2 studies) and children (6–10 years) (*n* = 2 studies), but the evidence suggests that LIPA does not influence mental health outcomes in these age groups.

**Conclusions:**

This review provided mostly cross-sectional evidence indicating that LIPA may not be associated with mental health outcomes across age groups. Future research efforts employing prospective research designs are warranted to better understand the role of LIPA on mental ill health across age groups.

**Supplementary Information:**

The online version contains supplementary material available at 10.1186/s12966-021-01196-7.

## Background

The burden of mental illness accounts for 32.4% of years lived with disability (YLDs) and 13.0% of lost disability-adjusted life-years (DALYs) globally [1]. Identifying modifiable risk factors and devising prevention strategies are crucial to reduce the burden of mental disorders. The recent World Health Organization (WHO) [2] guidelines on physical activity and the 2018 physical activity guidelines for Americans [3] acknowledge the benefits of engaging in regular physical activity for mental health (i.e., reduced symptoms of depression and anxiety). For such benefits, both sets of guidelines recommend that adults should regularly engage in at least 150–300 min of moderate-intensity aerobic physical activity; or at least 75–150 min of vigorous intensity aerobic physical activity (or an equivalent combination), and perform 2 days or more of muscle strengthening activities at least at moderate intensity [2, 3]. The majority of the evidence that has informed public health guidelines is derived from studies focused on moderate-to-vigorous physical activity (MVPA), and the extent to which light intensity physical activity (LIPA) contributes to mental health is less understood.

LIPA is an overlooked component of the physical activity continuum that refers to those activities that require an energy expenditure of 1.6–2.9 metabolic equivalents (MET) and usually include shuffling, indoor walking, household chores, occupational tasks, or incidental daily living movement [4]. Recently, the WHO Guidelines Development Group identified several research gaps in the existing literature including the need to conduct high quality research to examine the health benefits of LIPA across the lifespan [5]. As highlighted in current public health recommendations, “some physical activity is better than none” [2], and LIPA offers a great potential for increasing physical activity and overall energy expenditure [6]. Compared to MVPA which is mostly done during leisure-time, LIPA is inherently a larger component of waking times [7], and it may be the most acceptable form of physical activity since it mainly comprises unstructured movement that can easily be incorporated into everyday life. Engaging in LIPA may be a more enjoyable/pleasant way of staying active, while inactive people may be more likely to make long-term commitments to this type of physical activity. Additionally, increasing time spent in LIPA may also entail fewer potential barriers compared to higher physical activity intensities or more structured forms of exercise, which is likely to require more time, energy, skills, costs, facilities, and incur the risk of injury. Furthermore, several mental health conditions such as depression involve motivational/volitional deficits [8], and lack of motivation is a consistent predictor of moderate-to-vigorous intensity exercise [9]. Thus, given that LIPA may require less motivation as it is mostly accumulated though incidental daily living, it may be a key target to enhance behavior activation.

Several biological mechanisms have been proposed on how physical activity may reduce risk for mental health problems, and these include regulations in the hypothalamic–pituitary–adrenal axis, reduction in oxidative stress, anti-inflammatory activity, modulation of neurotransmitter release, regulations in the endogenous opioid system, stimulation of neurogenic processes, and changes in cortical activity and brain morphology [10–13]. It is currently unknown whether these mechanisms are driven solely by activity intensity or whether they could also be triggered by activity duration. Physical activity duration has been suggested to be a key factor for metabolic response [14, 15], and speculatively, it is possible that LIPA may be beneficial for mental health outcomes through the activation of duration-specific pathways. In addition, the relationship between physical activity and mental health is complex, and although very little is known specifically for LIPA, it may also benefit mental health indicators though a wide range of other psychosocial and behavioral pathways [16, 17].

To date, some systematic reviews have summarized the influence of LIPA on physical health outcomes such as cardiometabolic health and mortality [18–20], but to our knowledge, no comprehensive overview of the potential influence of LIPA on mental health has been published. A previous systematic review assessed associations of LIPA and health (including some mental health outcomes) [20]. However, due to the very narrow eligibility criteria (device-based measures of LIPA and confounding adjustment for MVPA), only one study with mental health indicators was included. A previous scoping review provided insightful evidence for the benefits of walking on mental health [21] but walking typically includes time that can also be characterized as moderate or even vigorous intensity physical activity [22, 23]. Considering the limited scope of the above reviews, synthesis of the evidence on LIPA and many aspects of mental health remains an important research gap.

Thus, the aim of our work was to systematically review and synthesize the observational evidence on the associations between LIPA and mental ill health in the general population. Mental ill health or negative indicators of mental health referred to the deleterious facets such as health problems and psychopathology [24]. To do that, we employed a life course approach integrating the literature across ages to determine whether these associations are more consistent at certain points of the lifespan. This approach takes into consideration differing levels and patterns of physical activity across age groups [25], and acknowledges the complex interplay of social and biological factors in the development of mental illness throughout the life-course [26].

## Methods

The current systematic review was conducted following the Preferred Reporting Items for Systematic Reviews and Meta-Analyses (PRISMA) guidelines to ensure comprehensive and transparent reporting (Additional file 1) [27] and was prospectively registered in PROSPERO database (ID CRD42020192834).

Systematic searches were performed in five electronic databases (Medline via PubMed, Embase, SCOPUS, PsychInfo, and CINHAL) from inception to May 28, 2020. The following search strategy was used: (light physical activity OR light-intensity physical activity OR low-intensity exercise OR lipa OR Lpa OR neat OR light exercise OR “non exercise activity thermogenesis” OR walking [MeSH] OR incidental physical activity OR incidental activity OR “lifestyle activity” OR “lifestyle physical activity”) AND (mental health [MeSH] OR depression OR anxiety OR negative affect OR psychological stress) AND humans. Additional manual searches were conducted using reference lists from recovered articles and relevant systematic reviews.

### Eligibility criteria

Observational studies (cross-sectional and longitudinal) published in English or Spanish were eligible for inclusion. Inclusion criteria were restricted to studies on the general population (healthy population without specific comorbidities, mental conditions or diseases). Studies measuring regular LIPA (either via self-report or through device-based measurements), defined as any physical activity of 1.6–2.9 MET were eligible. Studies were included if they reported mental ill health outcomes (depression, anxiety, psychological distress, perceived stress, negative affect, emotional problems, overall mental health) either as a primary or secondary outcome. Studies that targeted clinical populations, studies that examined the influence of acute single bouts of LIPA, and studies that investigated LIPA acting as the outcome were excluded. Articles assessing positive psychological outcomes (e.g., well-being, life satisfaction, positive affect) were excluded, as they were considered beyond the scope of the present review.

### Study selection

**Fig. 1:**
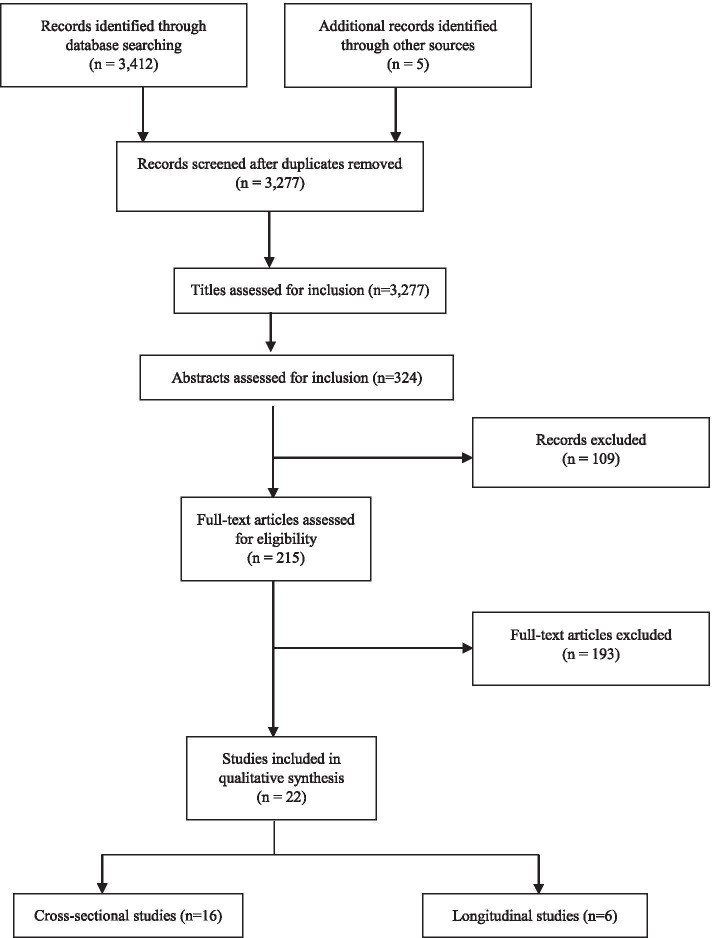
Preferred Reporting for Systematic Reviews and Meta-Analysis (PRISMA) diagram

Two reviewers (MF-N, JB) completed the identification of relevant records by title. After removal of duplicates, titles and abstracts were screened. Subsequently, full text articles were independently reviewed by MF-N and JB, and were assessed for inclusion (Fig. 1). A final list of included articles was developed though consensus, and a third reviewer (BS) was available for mediation in case of disagreement.

### Data extraction

ER, RM, MF and MF-N conducted data extraction with 80% checked for accuracy (MF-N). Extracted data included study characteristics (author, year, country, population characteristics), measurement tools of predictor and outcomes, and data describing the association between LIPA and mental health (if there were adjustments for confounders, only fully confounder-adjusted estimates are reported). For articles that reported results for several predictors and/or outcomes not exclusively related to LIPA and mental health, only data to address the question of interest was extracted. If relevant data were not included in the article, the corresponding author was contacted and asked to supply the data. If no answer was received after 1 month, a reminder was sent. If no answer was received after additional 2 weeks or if authors were unable to provide the requested data, the paper was included, and data is indicated as not reported. While the original aim was to provide a quantitative evidence synthesis through a formal meta-analysis, the structure of eligible literature did not permit this due to high heterogeneity in measures of LIPA and outcomes, and thus a narrative synthesis was conducted instead. The summary and discussion of the findings were conducted according to age groups (older adults, adults, young adults, adolescents, and children).

### Assessment of risk of bias

Study quality was reported using the Standard Quality Assessment Criteria for Evaluating Primary Research Papers from a Variety of Fields QUALSYST tool [28]. The Qualsyst score is based on 14 criteria such as question/objective sufficiently described, appropriate study design and research question, definition of outcomes and exposures, appropriateness of sample size, reporting of bias and confounding, and sufficient reporting of results and limitations. Criteria can be answered as ‘yes’ (2), ‘partial’ (1), ‘no’ (0), and ‘NA’ [28]. Included studies were independently rated by pairs of reviewers (ER and RM; JB and MF; BO and MF-N) and MF-N collated all information. In case of disagreement, consensus was reached via discussion and a more conservative approach was applied. A summary score was calculated for each paper by summing the total score obtained across relevant items and dividing by the total possible score. Items not applicable to a particular study were excluded from the calculation of the summary score [28].

## Results

### Study selection and characteristics of included studies

The search strategy identified 3,277 potentially relevant records from database searches. After applying inclusion and exclusion criteria, 22 studies were included in the review [29–50]. Main reasons for article exclusion were wrong definition/operationalization of LIPA, assessment of walking with no indicator of intensity (i.e., cadence), and outcomes unrelated to the focus of the present study. A flow diagram in Fig. 1 shows the study selection process and the characteristics of included studies are provided in Table 1. Of all included articles, 13 examined the association between PA intensities (including LIPA) with mental health indicators as a main research question, while in 8 studies, this was a secondary aim. Publication years of eligible articles ranged from 2009 to 2020. Of 22 included studies, 16 articles were cross-sectional [29–44] and 6 were longitudinal [45–50]. Studies were undertaken in a wide range of countries, with 3 studies conducted in upper middle-income countries (Lebanon, South Africa, China) and 19 in high-income countries or territories (US, UK, Japan, Hong Kong, Belgium, Taiwan, Ireland, Spain, Australia, Canada, Norway), [51] which accounts for 86.4% of total included articles. Studies reviewed were conducted in adults overall (18–64 years) [33, 34, 37–39, 48], older adults (≥ 65 years) [29–31, 45, 46], primary school children (6–10 years) [44, 50], adolescents (11–17 years) [43, 49], families (adolescents and adults) [42], and particular subgroups such as college students [40, 41], women from the university community (students and staff) [35], healthy pregnant women [36], and specific ethnic groups within a nation [32, 47]. There was substantial heterogeneity in the method of assessment of LIPA. Device-based measures were the most common tool to assess LIPA (*n* = 16), while some studies used self-reported assessments (*n* = 5) and 1 used both approaches. For device-based measures, accelerometry was used in 14 studies, step watch activity monitor in 1 study, the Actiheart in 1 study, and 1 study employed the activPAL. The most commonly used method to operationalize LIPA was counts per minute. Self-reported tools included the International Physical Activity Questionnaire (*n* = 2), the Pregnancy Physical Activity Questionnaire (*n* = 1), the Yale Physical Activity Survey (*n* = 1) and unvalidated questionnaires of physical activity. There was substantial heterogeneity in the method of assessment of the outcomes. Depression was measured with either the Patient Health Questionnaire (2 studies), the Geriatric Depression Scale (3 studies), the Center for Epidemiological Studies Depression Scale (4 studies), the Symptom CheckList-90 (1 study), the Zung Self-Rating Depression Scale (1 study), the Patient-Reported Outcomes Measurement Information System (1 study), or the Child Depression Inventory (1 study), while 1 study employed both the Computerized Clinical Interview Schedule-Revised and the Short Moods and Feelings Questionnaire at follow up. For anxiety, the State-Trait Anxiety Inventory was used in 2 studies, and different measures were used in all the rest of studies including the Symptom CheckList-90, the Hospital Anxiety and Depression Scale, and the Patient-Reported Outcomes Measurement Information System. Psychological distress was assessed with the General Health Questionnaire in 2 studies, and the Hopkins symptom checklist in another study. Overall mental health was assessed with a single item question on self-rated mental health; perceived stress via the Perceived Stress Scale; negative affect through the Positive and Negative Affect Scale; and emotional symptoms with the Strengths and Difficulties Questionnaire.

**Table 1:** Characteristics of cross-sectional and longitudinal studies grouped by age groups

**Author Year [ref]** **Design**	**Country**	**Participants sample size follow-up time**	**Measurement and operationalization of LIPA**	**Outcomes**	**Statistical analysis and effect size [95%CI]**	**Adjustment**	**Main findings**	**Quality score (%)**
Loprinzi 2013 [29] CS	US	Older adults (42.8% women; mean age = 73.5 years) *n* = 708	Accelerometry 100–2019 counts/min	Depression (Patient Health Questionnaire-9) [Score of ≥ 9 indicating depression]	Logistic regression Per 60 min increase OR = 0.80 [0.67, 0.95] *p* = 0.01	Age, gender, race-ethnicity, BMI, marital status, education, comorbidity index, physical functioning	LIPA was significantly associated with lower odds for depression.	90.9
Varma et al. 2014 [30] CS	US	Older adults (76.5% women; mean age = 66.8 years) *n* = 187	Step Watch Activity Monitor < 100 steps/min	Depressive symptoms (Geriatric Depression Scale)	Linear regression Per 1000 steps increase: β = -0.09 [-0.17, -0.01] Per 10 min increase: β = -0.02 [-0.05, -0.00] Number of bouts of 10 min activity): β = -0.04 [-0.07, -0.00]	Age, gender, race	Greater amount, frequency, and duration of LIPA were significantly associated with fewer depressive symptoms.	68.2
Yasunaga et al. 2018 [31] CS	Japan	Older adults (38.0% women; mean age = 74.4 years) *n* = 276	Accelerometry METs > 1.5 to < 3.0	Depressive symptoms (Japanese version of the 15-item Geriatric Depression Scale)	Linear regression Per 30 min increase β = -0.030 (-0.184, 0.124) Isotemporal substitution model β = -0.131 [-0.260, -0.002]	Gender, age, BMI, physical function, marital status, educational attainment, MVPA, sedentary behavior	LIPA (min/day) was not significantly associated with depression score Replacing 30 min per day of SB with the same amount of LIPA was significantly associated with lower depression score.	95.5
Ku et al. 2018 [45] LG	Taiwan	Older adults (54.4%; mean age = 74.5 years) *n* = 274 2 years	Accelerometry 100–1951 counts/min	Depressive symptoms (15-item Geriatric Depression Scale)	Linear regression RR = 0.67 [0.51, 0.88], *p* = 0.004	Sex, age, income source, drinking, number of diseases, insomnia, ADL difficulty, cognitive impairment, accelerometer wearing time, baseline depressive symptoms, MVPA	Participants who spent more time in LIPA had significantly fewer depressive symptoms at follow-up, independently of MVPA.	95.5
Uemura et al. 2017 [46] LG	Japan	Older adults (49.1% female; mean age = 71.5 years) *n* = 3,106 15 months	Self-reported unvalidated questionnaire (dichotomous variable)	Depression (15-item Geriatric Depression Scale) [Score of ≥ 6 indicating presence of depressive symptoms]	Logistic regressions Light exercise OR = 0.74 [0.56, 0.98] Walking habits OR = 0.76 [0.57, 1.01]	Age, gender, education, current smoking status, alcohol consumption, living status, self‐rated health, Mini‐Mental State Examination, Short Physical Performance Battery, medications, Geriatric Depression Scale at baseline	Engagement in light physical exercise but not walking was associated with significantly lower risk of depressive symptoms.	90.9
Ribeiro et al. 2017 [47] LG	US	Older African Americans (65.1% female; mean age = 66.1 years) *n* = 582 9 years	Self-reported Yale Physical Activity Survey	Depression (11-item version of the Center for Epidemiological Studies- Depression)	Logistic regression (Results not reported)	PA components (vigorous activity, leisure walking, moving, standing, and sitting), vegetable and fruit intake, age, gender, perceived income adequacy, years of formal education	Moving was not significantly associated with depression.	84.4
Rethorst et al. 2017 [32] CS	US	Hispanic/Latino community (female 52.1%; mean age = 41.1 years) *n* = 11,116	Accelerometry 100–1534 counts/min	Depressive symptoms (Center for Epidemiological Studies Depression Scale 10)	Linear regression β = 0.117 [-0.003, 0.237] *p* = 0.055 Isotemporal substitution model β = 0.003 [-0.011, 0.113] *p* = 0.955	Age, sex, Hispanic background group, BMI, household income level, education, recruitment site, physical health, general familial social support, acculturation, other PA intensities	LIPA (min/day) was not significantly associated with less depressive symptoms. Substitution of 1 h of SB with LIPA did not result in a significant decrease in depressive symptoms.	95.5
Asztalos et al. 2010 [33] CS	Belgium	Healthy adults (50.5% women; age 25–64 years) *n* = 6,803	Self-reported International Physical Activity Questionnaire [Dichotomized around 120 min/week]	Depression and anxiety (Symptom CheckList-90-Revised) [Mean score of ≥ 2 indicating depression or anxiety]	Logistic regression Men (depression) OR = 0.863 [0.631, 1.180] Men (anxiety) OR = 0.918 [0.644, 1.310] Women (depression) OR = 0.880 [0.700, 1.106] Women (anxiety) OR = 0.937 [0.723, 1.214]	Age, socioeconomic status	LIPA was not significantly associated with anxiety and depression in women and men.	86.4
Dillon et al. 2018 [34] CS	Ireland	Adults (53.9% female; mean age = 59.6 years) *n* = 397	Accelerometry dominant wrist = 191.8–281.5 counts/min non-dominant wrist = 159.5–261.8 counts/min	-Depression (Centre for Epidemiologic Studies Depression) -Anxiety (Hospital Anxiety and Depression Scale)	Isotemporal substitution models Anxiety: β = − 0.34 [-0.64, -0.04] Depression: β = -0.63 [-1.51, 0.26]	Age, gender, season, marital status, smoking status, alcohol consumption, BMI status, education	Substituting 30 min of SB for LIPA per day was associated with a significant decrease in levels of anxiety but not depression.	86.4
Poole et al. 2011 [35] CS	UK	Staff and student females (100% women; mean age = 28.7 years) *n* = 40	Accelerometry 191–573 counts/min	Depressive symptoms (Center for Epidemiologic Studies Depression Scale)	Pearson correlations *r* = -0.35 *p* < 0.05	None	Accelerometry-measured LIPA (min/day) was significantly correlated with fewer depressive symptoms.	54.5
Mourady et al. 2017 [36] CS	Lebanon	Healthy pregnant women (100% women; mean age = 30.5 years) *n* = 141	Self-reported Pregnancy Physical Activity Questionnaire	-Depression (Zung Self-Rating Depression Scale)	Spearman correlations Depression: *r* = -0.182. *p* = 0.031	None	Light PA (MET-hrs/week) has a significant inverse correlation with depression.	68.2
von Känel R et al. 2017 [37] CS	South Africa	Adults (52.2%women; mean age = 49.8 years) *n* = 203	Actiheart > 1.5- < 3METs	Psychological distress (28-item General Health Questionnaire)	Partial correlations (Results not reported)	Age, gamma glutamyl transferase	Partial correlations did not show any associations between PA and psychological distress.	63.6
Hamer M et al. 2014 [38] CS	UK	Adults (48.1–61.8% women; mean age = 48.3–50 years) *n* = 1,947	Accelerometry 200–2019 counts/min in tertiles	Psychological distress (12-item version General Health Questionnaire) [Score of ≥ 4 indicating psychological distress]	Logistic regression Highest tertile vs lowest OR = 0.73 [0.48, 1.12] Middle vs lowest tertile OR = 0.56 [0.37, 0.84]	Age, sex, accelerometry wear time, smoking, alcohol, education, BMI, social occupational group employment long-standing illness (non-mental), MVPA	LIPA was significantly associated with lower odds for psychological distress, independently of MVPA (although the association was not linear).	90.9
Sheikh et al. 2018 [48] LG	Norway	Adults (54% female, mean age = 47.0 years) *n* = 10,325 13 years	Self-reported. Unvalidated questionnaire [Categorical scale, none; less than 1; 1–2; 3 or more hrs/week]	Psychological distress (Hopkins symptom checklist, HSCL-10) [Score of ≥ 18.5 indicating psychological distress]	Regression analysis (ordinary least square and relative risk) β = 0.09 [-0.03, 0.22] RR = 1.03 [0.95, 1.11]	Age, gender, history of parental psychopathology, childhood socioeconomic status, marital status, daily smoking, number of friends, perceived social isolation, education, MVPA	LIPA did not confer significant protection against psychological distress at follow-up after adjusting for MVPA.	90.9
Bernard et al. 2018 [39] CS	Canada	Adults (50.3% women; mean age = 44 years) *n* = 8,150	Accelerometry 100 to 1534 counts/min	Overall mental health Single item question self-rated mental health	Generalized additive models LIPA = Estimate 7.2. f = 3. *p* = 0.003 LIPA + MVPA = Estimate 24.4. f = 3.6. *p* < 0.001 LIPA + SB = Estimate 21.6. f = 2.5. *p* < 0.001	Age, sex, daily smoking, household income, education level, accelerometer wear time, season of accelerometer assessment, BMI, MVPA	A curvilinear relationship between daily mins of LIPA and mental health was found, with better mental health found in 400–550 average mins of daily LPA.	90.9
Felez-Nobrega et al. 2020 [40] CS	Spain	College students (44% women; mean age = 20.8 years) *n* = 360 self-reported; *n* = 121 activPAL	Self-reported International Physical Activity Questionnaire [Tertile groups: T1 ≤ 3 h/week; T2 > 3 to ≤ 7 h/week; T3 > 7 h/week] Accelerometry %LIPAhrs/day based on time not spent standing, in MVPA or sedentary	-Perceived stress (Perceived Stress Scale) -Anxiety (State-Trait Anxiety Inventory)	Linear regression Self-reported LIPA: State anxiety: T2 vs T1 B = -1.70 [-5, 1.97]; T3 vs T1 B = -2.24 [-5.52, 0,86] Trait anxiety: T2 vs T1 B = -1,49 [-3.66, 0.90]; T3 vs T1 B = -1.47 [-3.51, 0.82] Perceived stress: T2 vs T1 B = -1.59 [-3.62, 0.46]; T3 vs T1 B = -2.07 [-3.90, -0.06] Partial correlation activPAL LIPA weekday; weekend day: State anxiety: *r* = 0.10, *p* = 0.69; *r* = -0.07, *p* = 0.51 Trait anxiety: *r* = -0.04, *p* = 0.64; *r* = -0.06, *p* = 0.54 Perceived stress: *r* = 0.04, *p* = 0.69; *r* = 0.07, *p* = 0.43	Age, gender	Self-reported LIPA was significantly associated with lower perceived stress but not with state-trait anxiety. No significant associations were found for device-based measures of LIPA (activPAL) with any of the mental health outcomes.	81.8
Tao et al. 2019 [41] CS	China	College students (52.3% women; mean age = 20.3 years) *n* = 220	Accelerometry Steps rate (steps/min) 20–99	Anxiety and depression (24-item Patient-Reported Outcomes Measurement Information System)	Pearson correlations Anxiety *r* = -0.023 (N.S) Depression *r* = 0.011 (N.S)	None	LIPA (min/day) was not significantly correlated with anxiety or depression.	72.7
Lee et al. 2013 [42] CS	Hong Kong	Non exercising healthy adolescents and adults (59.7% women; mean age = 46.2 years) *n* = 2,417	Accelerometry 101–1951 counts/min	Depression (Patient Health Questionnaire 9) [Score of ≥ 5 indicating depression (mild)]	Difference in mean z scores among those with and without depression -0.10 [-0.20, 0.01]	Age, sex	LIPA (min/day) was not significantly associated with depression.	95.5
Costigan et al. 2019 [43] CS	Australia	Healthy adolescents (44.9% girls; mean age = 12.9 years) *n* = 1,223	Accelerometry 101–2295 counts/min	Negative affect (the Positive and Negative Affect Scale for children)	Quantile regressions Standardized β = 0.010, *p* = 0.837	Sex, BMI, ethnicity, wear time, other physical activity intensities	No significant associations between LIPA and negative affect were found.	95.5
Kandola et al. 2020 [49] LG	UK	Healthy adolescents (56.1% girls; mean age 12 years) *n* = 4,257 6 years	Accelerometry 200–3599 counts/min	Depression Computerized Clinical Interview Schedule-Revised for depression at 18 years of age -The Short Moods and Feelings Questionnaire for depression at age 12, 14, and 16	Negative binomial regression models and by group-based trajectory modelling Increasing LIPA at age 12 (IRR = 0.904, [0.850, 0.961], *p* = 0.0012). Similar estimates were found for the other age groups	Sex, ethnicity, maternal social class, baseline depression, IQ, parental psychiatric history, parental education, total accelerometer wear time	At all timepoints, each 60 min/day increase in LIPA was significantly associated with a lower depression score at 18 years of age: 9.6% for LIPA at 12 years, 7.8% at 14 years, and 11.1% at 16 years of age. Lower depression scores were identified in participants with persistently high levels of LIPA.	100
Parfitt et al. 2009 [44] CS	UK	Healthy primary school children (59.6% girls; age 9–10 years) *n* = 57	Accelerometry Very LIPA = 100–470.1 counts/min LIPA = 470.1–976.8 counts/min	-Depression (Child Depression Inventory) -Anxiety (State-trait inventory for children)	Pearson correlations Very LIPA and anxiety *r* = 0.331. *p* < 0.05 Very LIPA and depression *r* = 0.282. *p* < 0.05 LIPA and anxiety *r* = 0.173. *p* = N.S LIPA and depression *r* = 0.202. *p* = N.S	Very light PA adjusted for percent body fat Correlations for LIPA not adjusted	Very LIPA (min/day) was significantly correlated with higher measures of anxiety and depression. No significant correlations were found for LIPA.	81.8
Ahn et al. 2018 [50] LG	UK	Children (61.3% female, mean age 11 years) *n* = 6,153 2 years	Accelerometry 100–2240 counts/min	Emotional symptoms (Strengths and Difficulties Questionnaire)	Linear regression Boys: β = -0.039 [-0.106, 0.028] Girls: β = -0.026 [-0.094, 0.041]	Age, season, total difficulties at age 7, limiting illness, special education needs, weight status, self-esteem, ethnicity, income, siblings, family structure, maternal education, maternal depression, maternal employment, British Ability Scale pattern construction, British Ability Scale word reading	LIPA was not significantly related to emotional symptoms at follow-up.	95.5

Only fully confounder-adjusted estimates are shown in tables

*Abbreviations*: *LIPA* Light intensity physical activity, *MVPA* Moderate-to-vigorous intensity physical activity, *PA* Physical activity, *min* Minutes, *BMI* Body mass index, *IQ* Intelligence quotient, *ADL* Activities of daily living, *SB* Sedentary behavior, *CS* Cross-sectional, *LG* Longitudinal

In terms of confounder adjustment, 3 studies (cross-sectional) only conducted univariable analysis when assessing relationships between LIPA and mental health indicators [35, 36, 41] and 6 studies (cross-sectional) only included few basic demographic variables [30, 33, 37, 40, 42, 44]. The rest of included studies (*n* = 13) adjusted for a variety of confounders with age, gender, BMI, ethnicity, education, income, marital status, and physical functioning being the most common control variables. Regarding adjustments for other movement related variables, 5 studies adjusted for MVPA [38, 39, 43, 45, 48], 2 studies adjusted for both MVPA and sedentary time [31, 32], and 1 study adjusted for vigorous activity, leisure walking, standing, and sitting [47].

The mean quality score for articles reporting cross-sectional data was 82.4%, with 31.3% of articles scoring below 75% (“relatively” conservative cut-off for acceptable articles [28]). Main reasons for lower scores were small sample size, robust measurement of outcome/exposures and lack of justification for analytic methods. Articles reporting longitudinal designs had higher quality scores with a mean score of 93.2%. The detailed quality scoring for each study can be found in the Additional file 2.

### Cross-sectional and longitudinal studies across the lifespan

Of the 16 articles that reported cross-sectional findings, 11 reported associations between LIPA and depression [29–36, 41, 42, 44], anxiety (5 studies) [33, 34, 40, 41, 44], psychological distress (2 studies) [37, 38], overall mental health (1 study) [39], perceived stress, and negative affect (1 study each) [40, 43]. Sample sizes ranged from *n* = 40 to 11,116. Most articles (*n* = 14, 87.5% of total) were based on device-based measures of LIPA (*n* = 10 hip/waist acceleromtery; *n* = 1 thigh; *n* = 1 ankle; *n* = 1 wrist; *n* = 1 Actiheart device). Longitudinal studies (*n* = 6) reported associations between LIPA and depression scores (4 studies) [45–47, 49], psychological distress (1 study) [48], and emotional problems (1 study) [50]. Sample sizes ranged from *n* = 274 to 10,325. Fifty percent of the studies assessed LIPA via device-based measures (waist/hip accelerometry) [45, 49, 50]. Follow-up assessments ranged from 2 to 13 years. Generally, the reported effect sizes for beneficial associations were small.

#### Older adults (≥ 65 years)

Beneficial associations between LIPA and depression scores/depressive symptoms were found in 2/3 cross-sectional studies [29, 30], 1/3 reported null associations [31], and beneficial associations were identified in the only study using isotemporal substitution models [31]. All studies used device-based measures of LIPA (placed at the hip/waist, and ankle) [29–31]. Only 1 study adjusted for MVPA [31]. For longitudinal designs, 1 large study (*n* = 3,106) [46] and 2 small studies [45, 47] assessed the association of LIPA with subsequent depressive symptoms in older adults. Significant associations were reported in 2/3 studies (one of these studies adjusted other PA intensities). One study assessed LIPA via waist accelerometry [45] and the others via self-reported measures [46, 47]. No evidence was found for other mental health indicators in older adults.

#### Adults (18–64 years)

For the association between LIPA and depression scores/depressive symptoms, fairly consistent evidence (3/5 studies) was found. LIPA was unrelated to depressive symptoms either in cross-sectional or isotemporal substitution models [32–34]. The two studies based on unadjusted correlations (2/5) that found beneficial associations were among young women from the university community and in healthy pregnant women, and assessed LIPA via hip accelerometry and via self-report [35, 36].

Two large cross-sectional studies in adults reported on anxiety and showed divergent findings. No statistically significant associations were reported in 1 cross-sectional study that used self-reported measures of LIPA [33], while the wrist accelerometry based isotemporal substitution study reported beneficial associations [34].

Three studies (2 cross-sectional and 1 longitudinal) examined relationships between LIPA and psychological distress [37, 38]. Cross-sectional studies used device-based measures for assessing LIPA (Actiheart and waist accelerometry), and although the smaller study (*n* = 203) found no associations, [37] significant and beneficial associations were reported by the larger (*n* = 1,947), after adjusting for common confounders and MVPA [38]. Furthermore, self-reported LIPA did not show beneficial associations with psychological distress at follow-up, according to a large study (*n* = 10,325) in adults after adjustment for MVPA [48].

One large study (*n* = 8,150) that used hip accelerometry investigated the link between LIPA and a measure of overall mental health and found significant beneficial associations [39]. Nonetheless, the study also reported that a LIPA dose ranging from 200 to 350 min was associated with a low mental health level, regardless of MVPA dose.

#### Young adults (college students)

Two studies examined the association of LIPA and anxiety, and null findings were observed in both, independently of the tools used for LIPA assessment (thigh and hip accelerometry) [40, 41].

We identified only 1 study focusing on associations between LIPA and perceived stress in college students [40]. The study reported divergent findings depending on the PA measurement tool identifying beneficial associations only for self-reported measures of LIPA [40]. No adjustments for MVPA were included.

#### Adolescents (11–17 years)

In adolescents, we only identified 1 cross-sectional study assessing the relationship between hip accelerometry-based LIPA and depression, and this reported non statistically significant associations [42]. However, adults were also included in the sample. One large longitudinal study (*n* = 4,257) was identified for depression and reported that increases in hip accelerometry-measured LIPA were associated with a lower depression score at follow up [49]. No adjustments for MVPA were conducted.

Finally, 1 study that assessed relationships between waist accelerometry-based LIPA and negative affect while taking into account of other PA intensities reported no significant relationships [43].

#### Children (6–10 years)

We only found 1 cross-sectional study in children assessing the relationships between hip accelerometry-based LIPA and depression and anxiety [44]. This study reported null findings for LIPA but found that very LIPA (100–470.1 counts/minute) was significantly correlated with higher depressive symptoms and anxiety [44]. No adjustments for other PA intensities were included in the analysis.

One large longitudinal study assessed the relationship of waist accelerometry-based LIPA and emotional problems, and no statistically significant associations were reported [50]. No adjustments for MVPA were conducted.

## Discussion

To our knowledge, this is the first systematic review that provides some initial insights on the relationship between LIPA and mental health indicators. We identified a limited number of studies across age groups, and a high level of heterogeneity in LIPA measurements and mental ill health outcomes assessed. In addition, important methodological weaknesses in the literature were found. Findings on the relationship between LIPA and mental ill health indicators were mixed across all age groups but overall, there seems to be limited evidence suggesting that LIPA benefits mental ill health indicators.

In an attempt to enlighten and disentangle the potential reasons underlying the inconsistent findings, we compared cross-sectional and longitudinal results in the studies that reported the same outcomes in the same population (i.e., depressive symptoms in older adults; psychological distress in adults). For depression/depressive symptoms, high quality cross-sectional studies in older adults found that LIPA was significantly associated with lower depressive symptoms [29, 31], and this was consistent with most high quality longitudinal studies [45, 46]. However, caution is urged when interpreting these results since these studies had small sample sizes and most of them did not adjust for MVPA. Furthermore, for psychological distress, the evidence derived from cross-sectional and longitudinal studies is conflicting. One high quality cross-sectional study found that LIPA was associated with reduced psychological distress [38], while another high quality longitudinal study showed null relationships [48]. Both studies adjusted for MVPA but this later study used a self-reported measure of LIPA with no previous evidence of validity or reliability [48]. Improvements in research design and more longitudinal research will allow for a more profound understanding on the relationship between LIPA and mental ill indicators across populations. Relatedly, an important methodological weakness found in the current literature is that some studies used correlational methodologies that cannot provide any evidence on causation, or employed small samples.

Another potential explanation for inconsistencies in findings may be related to the lack of appropriate control variables. For instance, only a few studies adjusted the analysis for MVPA, which may have been a residual confounder (older adults, *n* = 4/6 studies; adults, *n* = 4/9; young adults, *n* = 0/2; adolescents *n* = 1/4; children *n* = 0/1). While we did not find a consistent direction of findings when comparing between those studies that did and did not adjust for MVPA, taking MVPA into account is important because people may be engaged in both behaviors throughout the day, and beneficial associations between LIPA and mental health indicators may be confounded by MVPA. Similarly, some studies in older adults adjusted the association between LIPA and depression for other PA components and/or sedentary behavior [31, 47]. While significant associations were found in the studies that only adjusted for a few confounders (which may also include MVPA) [29, 30, 45, 46], non-significant associations were reported in those that included more physical activity components or sedentary behavior.

Additionally, other factors such as the context/domain in which LIPA occurs may be crucial to unravel associations with mental health. In this regard, previous evidence indicates that some domains are more important in promoting mental health and preventing mental illness than others (such as leisure-time physical activity, transportation, school sport) [52]. Furthermore, it might be possible that, rather than the volume of LIPA, other psychosocial and behavioral correlates and determinants of physical activity is what really matters for mental health (e.g., how LIPA was undertaken, its purpose, with whom, natural environment, self-efficacy). Similarly, personality traits and genetics may be important, since they are known to explain a significant portion of the variance in mental health outcomes [53, 54]. Unfortunately, little is known about how these factors may influence the relationship between LIPA and mental health as such factors are rarely accounted for.

When focusing on studies that employed device versus self-reported instruments for assessing LIPA, no consistent associations were found. LIPA measurement presents some serious challenges that may be inherent to this construct per se and limit the ability to make sound interpretations from the evidence. Most included studies assessed LIPA via accelerometry, but there is limited agreement regarding the optimal waist accelerometry cut-off points for LIPA for the different age groups [55]. An undoubtedly more challenging issue is the use of self-reported instruments (used in 5/22 included studies). Their ability to capture LIPA in a valid and accurate manner is very limited since recall challenges the ubiquitous presence and sporadic nature of lower physical activity intensities [56]. Advances in device-based measurement of LIPA would provide useful insights to better understand its relationship with mental health. For instance, it is possible that different LIPA ranges may offer graded benefits across the whole physical activity continuum.

Finally, it seems that LIPA is not associated with mental ill health indicators consistently across all age groups and across all mental ill health indices included in the current study. Given that the evidence on the relationship between mental health and LIPA is in its incipient stages, more high-quality studies are needed to determine whether LIPA is selectively associated with specific mental ill health indicators and whether the magnitude of the association is greater at some ages relative to others.

### Strengths and limitations

The current study offers novel findings in proving a first synthesis of the evidence regarding the association between LIPA and mental health across the lifespan. The main limitation of this review is the small amount of evidence found and its heterogeneity. Moreover, publication bias, limiting the generalizability of our findings, may be present, but this limitation is inherent in all systematic reviews. Due to the relative novelty of this line of research, intervention studies were not included in the present review, and thus, present findings limit the ability to draw causal conclusions. Additionally, given that the largest evidence base was cross-sectional, a bi-directional association is also possible for LIPA and mental health. Finally, a limitation of the current study is that we did not include positive psychological outcomes. There is evidence indicating that MVPA is associated with positive psychological mental health outcomes [57], and the influence of LIPA on these indicators should also be examined in future research endeavors.

## Conclusions

This review provided evidence indicating that LIPA may not be associated with mental ill health outcomes across the lifespan. Due to its proven benefits on several mental health indicators, regular engagement in MVPA should be encouraged in the first instance. However, LIPA may be a more compelling approach to foster a physically active lifestyle in those population groups where MVPA is less feasible such as older people or frail populations. Future research efforts employing more rigorous methodologies and stronger research designs are warranted to better understand the role of LIPA on mental health across age groups. In addition, a transdisciplinary approach accounting for several biopsychosocial factors will help to better understand the complex relationship between physical activity and mental health.

## Supplementary Information

**Additional file 1.** PRISMA 2020 Checklist.

**Additional file 2.** Detailed quality scoring for each study.

## Data Availability

Authors can confirm that all relevant data are included in the article.

## Abbreviations

LIPA: Light intensity physical activity
MVPA: Moderate-to-vigorous intensity physical activity
PA: Physical activity
BMI: Body mass index
IQ: Intelligence quotient
ADL: Activities of daily living
SB: Sedentary behavior
CS: Cross-sectional
LG: Longitudinal
